# The effect of L‐arginine supplementation on maximal oxygen uptake: A systematic review and meta‐analysis

**DOI:** 10.14814/phy2.14739

**Published:** 2021-02-15

**Authors:** Shahla Rezaei, Maryam Gholamalizadeh, Reza Tabrizi, Peyman Nowrouzi‐Sohrabi, Samira Rastgoo, Saeid Doaei

**Affiliations:** ^1^ Student Research Committee Department of Clinical Nutrition School of Nutrition and Food Sciences Shiraz University of Medical Sciences Shiraz Iran; ^2^ Nutrition Research Center School of Nutrition and Food Sciences Shiraz University of Medical Sciences Shiraz Iran; ^3^ Student Research Committee, Cancer Research Center Shahid Beheshti University of Medical Sciences Tehran Iran; ^4^ Non‐Communicable Diseases Research Center Fasa University of Medical Sciences Fasa Iran; ^5^ Department of Biochemistry, School of Medicine Shiraz University of Medical Sciences Shiraz Iran; ^6^ Department of Clinical Nutrition and Dietetics National Nutrition and Food Technology Research Institute Shahid Beheshti University of Medical Science Tehran Iran

**Keywords:** L‐arginine, maximal oxygen uptake, meta‐analysis, VO2 max

## Abstract

**Background:**

The efficacy and safety of L‐arginine supplements and their effect on maximal oxygen uptake (VO2 max) remained unclear. This systematic review aimed to investigate the effect of L‐arginine supplementation (LAS) on VO2 max in healthy people.

**Methods:**

We searched PubMed, Scopus, Web of Science, Cochrane, Embase, ProQuest, and Ovid to identify all relevant literature investigating the effect of LAS on VO2 max. This meta‐analysis was conducted via a random‐effects model for the best estimation of desired outcomes and studies that meet the inclusion criteria were considered for the final analysis.

**Results:**

The results of 11 randomized clinical trials indicated that LAS increased VO2 max compared to the control group. There was no significant heterogeneity in this meta‐analysis. Subgroup analysis detected that arginine in the form of LAS significantly increased VO2 max compared to the other forms (weighted mean difference = 0.11 L min^−1^, *I* ^2^ = 0.0%, *p* for heterogeneity = 0.485).

**Conclusions:**

This meta‐analysis indicated that supplementation with L‐arginine could increase VO2 max in healthy people. Further studies are warranted to confirm this finding and to identify the underlying mechanisms.

## Abstract 2

This meta‐analysis indicated that the supplementation of L‐arginine could increase VO2 max in healthy people

## 1 INTRODUCTION

Cardiovascular endurance is one of the most important measures of overall health (Ruiz et al., 2006). A person's level of cardiovascular endurance helps predict ability to react to acute physical and mental stress (Gutin et al., 2005). For healthy individuals, higher cardiovascular endurance also can indicate an elevated level of physical fitness (Haghshenas et al., 2019). One of the best indicators for the athlete's cardiovascular performance is the maximal oxygen uptake (VO2 max) assessment (Campbell et al., 2006). A greater amount of oxygen consumed by the body is related to higher cardiovascular efficiency (Adams et al., 1995). Higher cardiovascular efficiency allows muscle to work at a higher intensity for a longer time period. The body can only exercise as long as oxygen is delivered to the muscle and waste products such as carbon dioxide are removed (Haghshenas et al., 2019). Many factors such as proteins could be associated with cardiovascular risk factors and other diseases (Doaei et al., 2018; Shidfar et al., 2018).

Amino acids are among the most common nutritional supplements which are used by athletes to improve athletic performance under aerobic and anaerobic conditions (Mashiko et al., 2004). L‐arginine is one of the semi‐essential amino acids that has positive effects on muscle metabolism (Preli et al., 2002). L‐arginine may also have a key role in the cardiac function of athletes. Arginine is a precursor of nitric oxide (NO) and NO causes vasodilatory effects, increased blood flow to the muscles, and increased release of certain hormones such as insulin and human growth hormone (Adams et al., 1995; Moazami et al., 2015). Oral L‐arginine supplements improved coronary endothelium‐dependent dilation (Melik et al., 2017).

L‐arginine may have led to delayed fatigue by altering blood lactate concentration and metabolic indices of respiration. It is frequently reported that using L‐arginine supplement may improve athletic performance in sports activities. (Yaman et al., 2010). Yaman et al. found that L‐arginine supplementation (LAS) significantly reduced blood pressure and increased VO2 max and may influence athletic performance capacity (Kalman et al., 2016).

However, the studies on the association between LAS and VO2 max reported contradictory results. Therefore, this systematic review and meta‐analysis aimed to investigate the effect of LAS on VO2 max.

## 2 METHODS AND MATHERIALS

### 2.1 Literature search strategy

This systematic review and meta‐analysis was performed in accordance with PRISMA (Preferred Reporting Items for Systematic Reviews and Meta‐Analyses) guidelines (Liberati et al., 2009). The scientific databases including PubMed, Scopus, Web of Science, Cochrane, Embase, ProQuest, and Ovid were reviewed to identify all relevant literature on the effects of LAS on VO2 max that were published by August 2020. The following search strategy was used to finalize the first step of data gathering: (Arginine[Mesh] OR Arginine[tiab]) AND (VO2[tiab] OR "maximal aerobic"[tiab] OR "aerobic capacity"[tiab] OR "maximal O2"[tiab] OR "maximal O2 consumption"[tiab] OR "maximal O2 uptake"[tiab] OR "peak O2"[tiab] OR "maximal oxygen consumption"[tiab] OR "maximal oxygen uptake"[tiab] OR "peak oxygen uptake"[tiab] OR "maximal aerobic capacity"[tiab]).

To enhance the quality of the searches, hand searching was performed to find all relevant articles using the references of the collected articles. The searches were limited to human studies and no language restriction was used in the search process. Two authors (Sh. R and P. N) independently screened the title and the abstracts of the included papers, performed data extraction, and carried out the quality assessments of the eligible studies. All disagreements were resolved by consulting with a third author (R. T).

### 2.2 Study selection

The following strategy was used to select the eligible papers for performing the meta‐analysis: Randomized clinical trials (parallel or cross‐over) experiments, investigated the effect of LAS on VO2 max in healthy human subjects, individuals supplemented with arginine were compared to placebo‐control individuals, arginine supplementation administered for at least 1 week, papers with enough information to measure the VO2 max, papers contained data for SD, SE, and CI parameters in the beginning and the end of the study for both of the intervention and control groups.

### 2.3 Data extraction

All eligible randomized controlled trials were separately re‐checked and the following data were extracted: the name of the first author, country, the number of individuals in the intervention and control groups, the form of supplemented arginine, arginine doses, duration of the study, type of the study, and the related data for further steps (Table 1). For each study, the value of mean and SD for VO2 max in the beginning and at the end of the study was extracted. The following formula was used to calculate the mean difference of SDs:

$$\begin{array}{cc} \text{SD} & =\text{square}\;\text{root}\left[{\left(\text{SD}\;\text{at}\;\text{baseline}\right)}^{2}+{\left(\text{SD}\;\text{at}\;\text{the}\;\text{end}\;\text{of}\;\text{study}\right)}^{2}\right)\\ & \;\left(‐(2R\times \text{SD}\;\text{at}\;\text{baseline}\times \text{SD}\;\text{at}\;\text{the}\;\text{end}\;\text{of}\;\text{study})\right]. \end{array}$$

**TABLE 1:** Participant and intervention characteristics of the studies included in the systematic review and meta‐analysis

ID	Authors	Year	Country	Population	Age	Number of subjects in intervention/control groups	Type of study	Type of Intervention	Control group	Duration of study
1	Camic et al.	2010	USA	College‐aged male	22.1 ± 2.4	21/20	Randomized, double‐blind, placebo, controlled, parallel design	3 g/day, LAS+300 mg of grape seed extract + 300 mg of polyethylene glycol	Placebo	28 days
2	Chen et al.	2010	USA	Male cyclists	ARG:57.6 ± 4.6 PLA: 60.6 ± 8.7	8/8	Two‐arm prospectively randomized double‐blinded and placebo‐controlled trial	5.2 g/day, LAS + L‐citrulline + 500 mg ascorbic acid, 400 IU vitamin E, 400 μg folic acid, 300 mg L‐taurine, and 10 mg alpha lipoic acid	Placebo	21 days
3	Muazzezzaneh et al.	2010	Iran	Healthy athletes	22.66 ± 1.46	14/13	Based on a single‐blind placebo‐controlled trial	5 g/day, LAS	Placebo	21 days
4	Sunderland et al.	2011	USA	Endurance‐trained male cyclists	36.3 ± 7.9	9/9	Randomized, conducted in a double‐blind manner	12 g/day, LAS	Placebo	28 days
5	Moazami et al.	2014	Iran	Female handballists	2.49 ± 22.15	8/8	Randomized clinical trial	3 g/day, LAS	Placebo	7 days
6	Zak et al.	2015	USA	Untrained men	22.0 ± 1.7	19/19	Double blinded, placebo‐controlled, within subjects’ crossover design	3 g/day, LAS + 300 mg of grape seed extract (95% procyanidins), and 300 mg of polyethylene glycol	Placebo	7 days
7	Hosseini et al.	2015	Iran	Healthy futsal players	22.5 ± 1.39	10/10	Randomized control trial	4 g/day, LAS	Placebo	28 days
8	Pahlavani et al.	2017	Iran	Soccer players	20.85 ± 4.29	25/27	Double‐blinded, randomized, placebo‐controlled trial	2 g/day, LAS	Placebo	45 days
9	Dennis et al.	1991	France	Medical students, active in recreational activities	19–26	15/15	Double‐blind, cross‐over study	5 g/day, AA	Placebo	10 days
10	Burtscher et al.	2005	Austria	Healthy athletes	22 ± 3	8/8	Double blind placebo‐controlled trial	3 g/day, AA	Placebo	21 days
11	Campbel et al.	2005	USA	Resistance‐trained healthy adult men	38.9 ± 5.8	20/15	Randomized, double‐blind, controlled design	12 g/day, AAKG	Placebo	56 days

A correlation coefficient of 0.5 was used for *R*, estimated between 0 and 1 values, respectively. Also, the formula $\mathrm{SD}=\mathrm{SE}\times \sqrt{n}$ (*n* = the number of individuals in each group) was used to measure SD in each article that reported SE instead of SD.

### 2.4 Quality assessment

**FIGURE 1:**
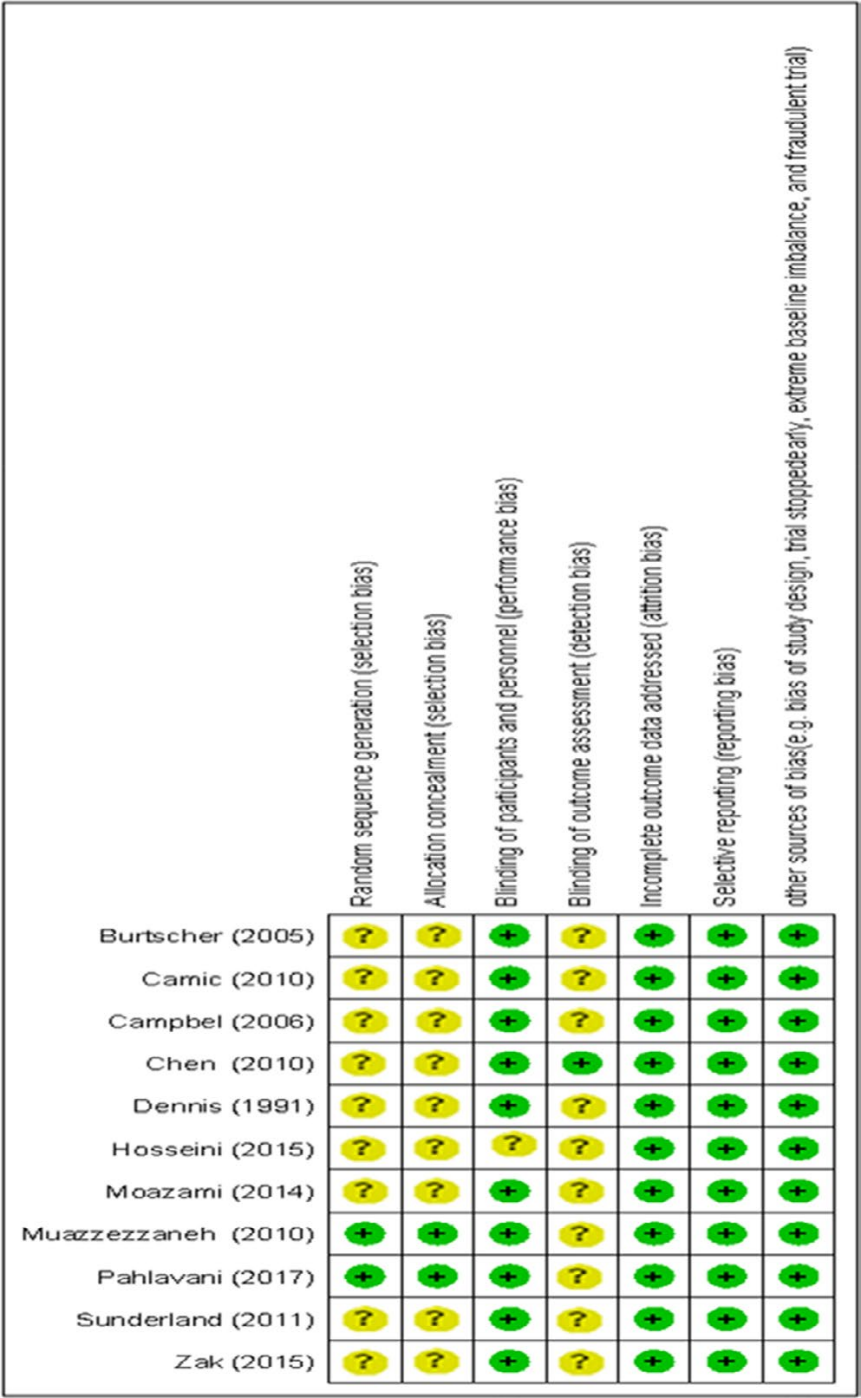
Summary of risk of bias: According to Cochrane criteria, any source of bias including selection bias, performance bias, detection bias, attrition bias, and reporting bias were judged for all included studies

The quality assessment of the included papers in this meta‐analysis was performed according to Cochrane criteria (Higgins, 2011). According to this guideline, any source of bias including selection bias, performance bias, detection bias, attrition bias, and reporting bias were judged for all included studies (Figure 1).

### 2.5 Statistical analysis

**FIGURE 2:**
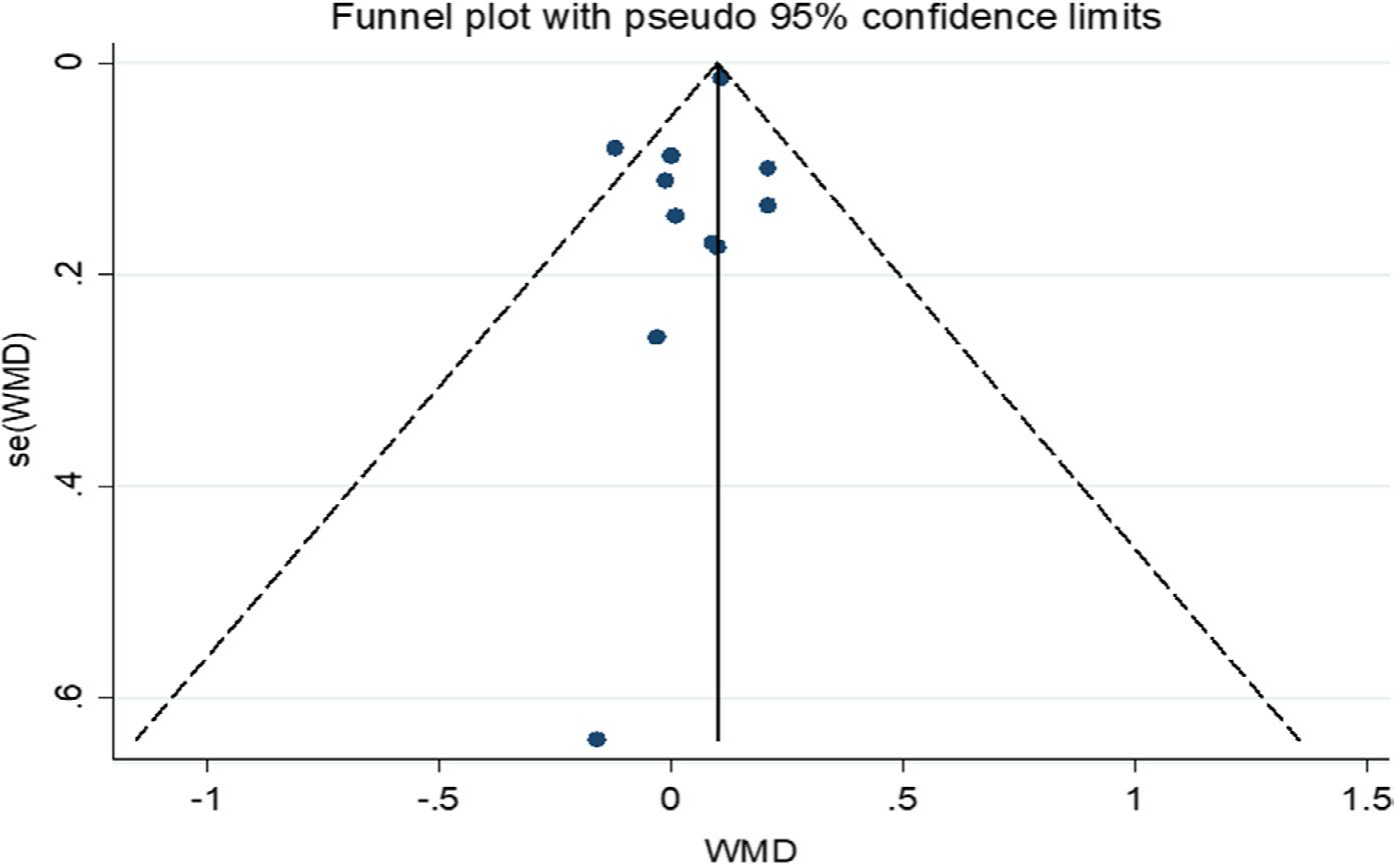
Publication bias was checked by the funnel plot, Begg's (*p* = 0.815) test, and Egger's (*p* = 0.218) tests. SE, standard error; WMD, weighted mean difference

This meta‐analysis was conducted using Stata version 11. Due to the population selection from different countries, a random‐effects model was employed with a 95% confidence interval (CI) for the calculation of the pooled weighted mean difference (WMD). Analysis endpoints were calculated as the difference in mean between baseline and post‐treatment (measure at the end of follow‐up − measure at baseline); also, the SD of mean change was calculated by the pooled SD. The statistical heterogeneity between trials was calculated by p‐value and using I2 statistic (*p* < 0.05 and *I* ^2^ > 50%). Publication bias was checked by the funnel plot, Begg's test (*p* = 0.815), and Egger's tests (*p* = 0.218; Figure 2).

## 3 RESULTS

### 3.1 Search results and study selection

**FIGURE 3:**
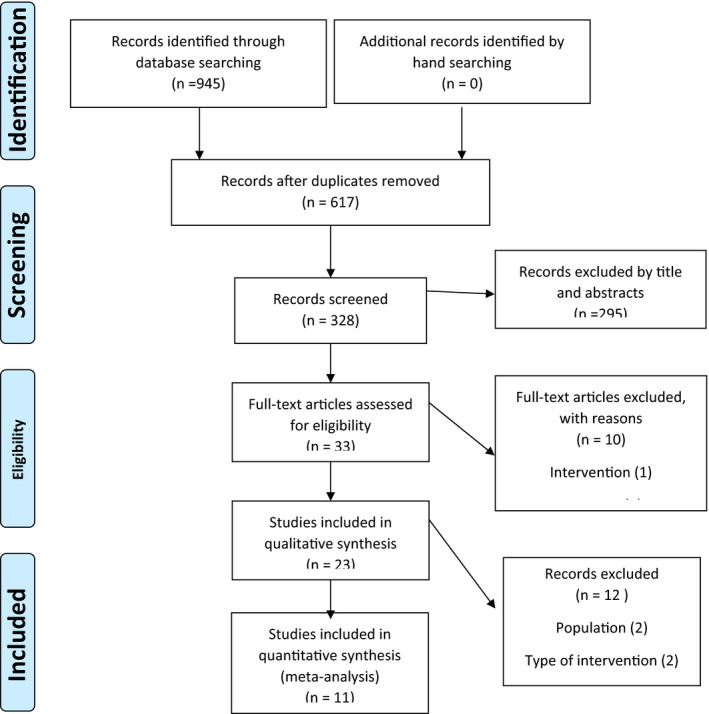
Preferred Reporting Items for Systematic Review and Meta‐Analyses flow diagram

The flow chart presented in Figure 3 describes the process of selection and the references retrieved in the database. A total number of 945 articles was identified in the first step of the literature search of electronic databases. After excluding duplicated studies (*n* = 617), irrelevant studies based on title and abstracts (*n* = 295), type of intervention (*n* = 1), type of outcomes (*n* = 5), and the required data (*n* = 4), 23 potentially relevant articles were considered for full text review. After screening, 12 articles were excluded for the following reasons: studies population, insufficient data reporting of outcome, and type of LAS. Finally, 11 studies were included in the present meta‐analysis (Burtscher et al., 2005; Camic et al., 2010; Campbell et al., 2006; Chen et al., 2010; Denis et al., 1991; Hosseini et al., 2015; Moazami et al., 2015; Muazzezzaneh et al., 2010; Pahlavani et al., 2017; Sunderland et al., 2011; Zak et al., 2015).

### 3.2 Quantitative data synthesis

**FIGURE 4:**
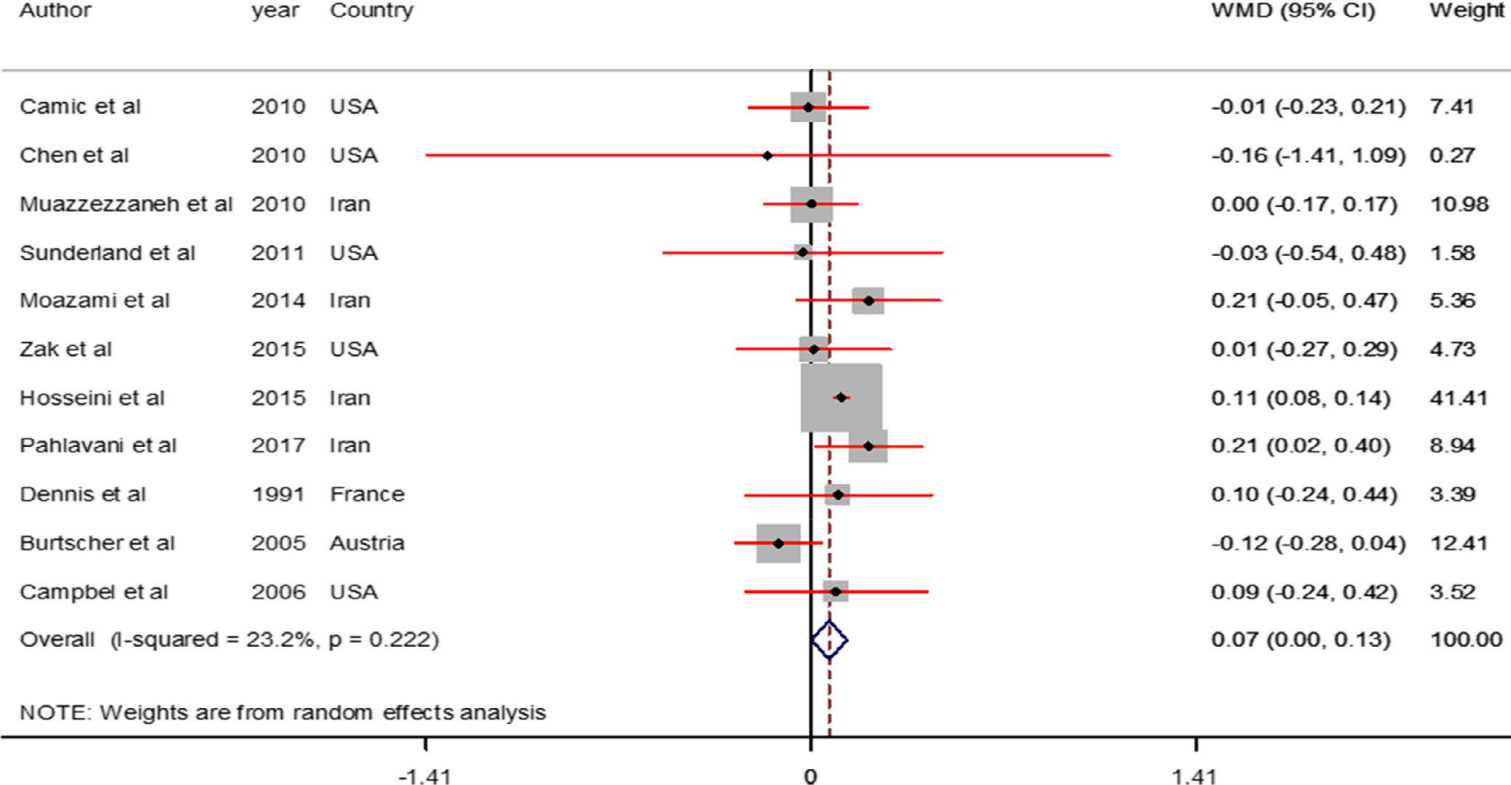
Forest plot comparing the effects of L‐Arg supplementation on VO2 max

Marginal significant increase in VO2 max (WMD = 0.07 L min^−1^; 95% CI, 0.00–0.13, *p* = 0.047; *I* ^2^ = 23.2%) was found after L‐ arginine supplementation in comparison with the control group (Figure 4).

### 3.3 Subgroup analysis

Subgroup analysis was performed based on the study duration (≥14 days), dosage of L‐arginine (<5 g/day), and the type of arginine supplementation including LAS, arginine aspartate, arginine alpha‐Ketoglutarate, and arginine in combination with antioxidants to detect the source of heterogeneity. There was a significant increase in VO2 max in the subgroup analysis of trials with LAS (WMD = 0.11 L min^−1^, *I* ^2^ = 0.0%, *p* for heterogeneity = 0.485; Table 2).

**TABLE 2:** Subgroup analysis was performed based on the study duration, dosage of L‐arginine, and the form of arginine supplementation

Subgroup analysis for VO2 max					
Subgroup	No. of trials	WMD (95% CI)	Test for the overall effect	Test for heterogeneity	*I* ^2^ (%)
Duration of study (days)					
˃14 days	8	0.05 (−0.04, −0.13)	*p* = 0.261	*p* = 0.102	41.5
≤14 days	3	0.11 (−0.06, 0.28)	*p* = 0.188	*p* = 0.596	0.0
L‐arginine dose (g/day)					
<5	6	0.07 (−0.03, 0.17)	*p* = 0.186	*p* = 0.047	55.4
≥5	5	0.03 (−0.11, −0.16)	*p* = 0.704	*p* = 0.969	0.0
Type of L‐arginine					
LAS	5	0.11 (0.08, 0.14)	*p* = 0.000	*p* = 0.485	0.0
AA	2	−0.06 (−0.25, 0.12)	*p* = 0.506	*p* = 0.250	24.5
Other	4	0.01 (−0.14, 0.17)	*p* = 0.852	*p* = 0.956	0.0

Abbreviations: AA, arginine aspartate; AAKG, arginine alpha‐Ketoglutarate; LAS, L‐arginine supplementation.

### 3.4 Sensitivity analysis

**FIGURE 5:**
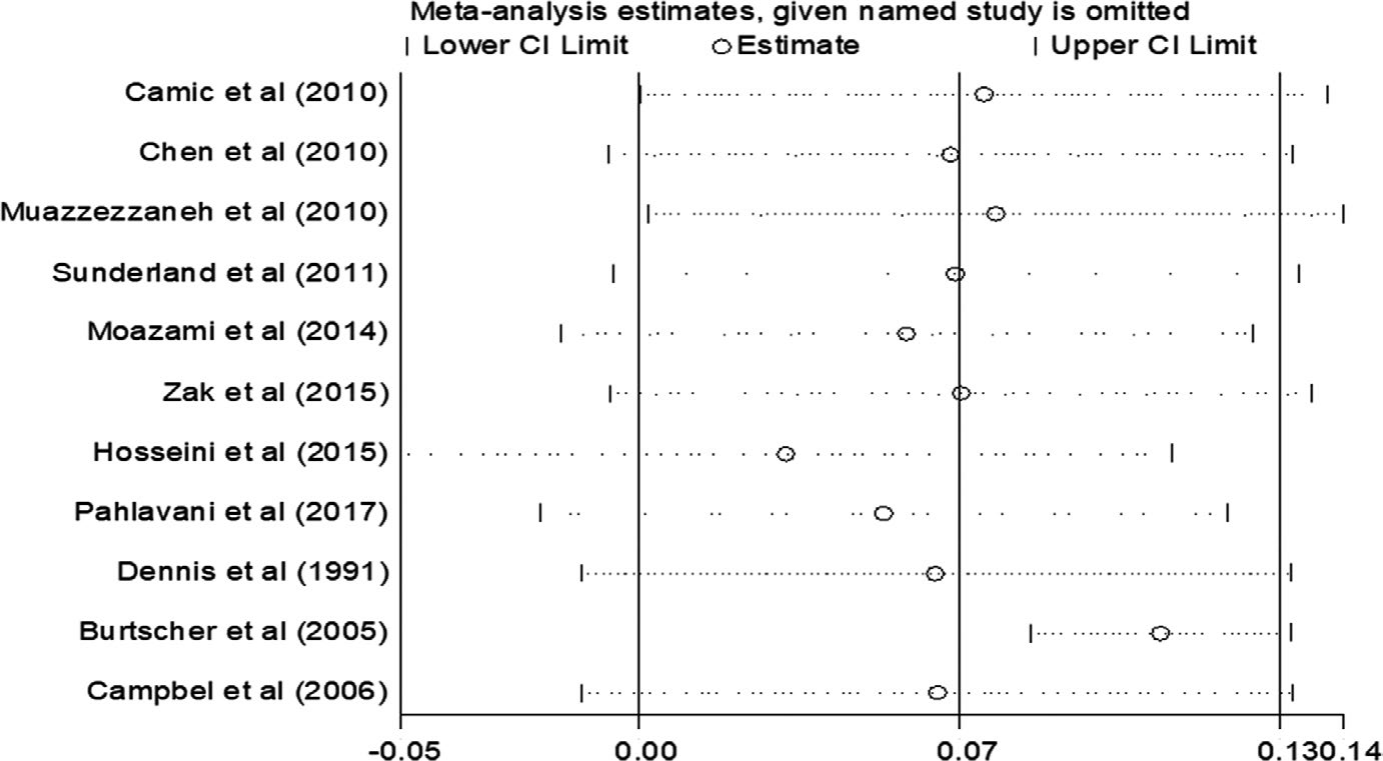
The sensitivity analysis was performed using the “one‐study‐removed” method to survey the impact of each study on the effect size

The sensitivity analysis was performed using “one‐study‐removed” method to survey the impact of each study on the effect size. The results of sensitivity analysis identified the higher and lower pooled weight mean difference for VO2 max (WMD = 0.1 L min^−1^; 95% CI 0.08, 0.13) after excluding the Burtscher et al. (2005) study and (WMD = 0.03 L.min^‐1^; 95% CI 0.04, 0.1) after excluding Hosseini et al. (2015) study, respectively (Figure 5).

## 4 DISCUSSION

This is the first meta‐analysis that evaluates the effect of LAS on VO2 max in healthy human subjects. The results indicated that LAS resulted in a mean increase of 0.07 L min^−1^ for VO2 max compared with placebo (95% CI, 0.00–0.13). No significant heterogeneity was detected in this meta‐analysis. The subgroup analysis indicated that supplementation with L‐arginine alone significantly increased VO2 max compared to the other types of arginine or combined with other metabolites or supplements.

Evidence suggest the relationship between LAS and improved exercise performance. L‐arginine is reported to have a key role in creatine synthesis as well as in increase endogenous growth hormone (Campbell et al., 2004). L‐arginine is also the substrate for nitric oxide synthesis that plays a role in the autoregulation of blood flow, myocyte differentiation, respiration, and glucose homeostasis in muscle (Stamler & Meissner, 2001). Although some studies have shown a positive effect of LAS on exercise performance, the results of the trials which assessed the effect of LAS on VO2 max were inconsistent.

A positive effect of LAS on VO2 max was identified in the present meta‐analysis. This finding is generally in line with some of the individual studies selected for this review. Hosseini et al. (2015) reported that 4 g/day arginine supplementation for 4 weeks could significantly increase VO2 max and subsequently improved sports performance in athletes. Another study conducted by Moazami et al. (2015) reported that VO2 max was significantly increased after a 7‐day supplementation of L‐arginine at the dose of 21 g/day in female athletes. In addition, a placebo‐controlled trial (Pahlavani et al., 2017) indicated that the oral supplementation of L‐arginine at the dose of 2 g/day for 45 days could increase VO2 max. Conversely, Burtscher et al. (2005) found that 3 weeks of L‐arginine‐L‐aspartate supplementation at the dose of 3 g/day resulted in lower oxygen consumption and reduced ventilation during submaximal cycle exercise. This may be explained by the difference in physiological functions at a maximum level of effort compared with a submaximal (Larsen et al., 2007). It seems that nitric oxide derived from L‐arginine through competitive inhibition of oxygen use in the electron transport chain result in lower whole‐body oxygen consumption at submaximal work (Burtscher et al., 2005; Larsen et al., 2007; Schweizer & Richter, 1994).

However, some studies did not observe any significant association between the intake of LAS and VO2 max (Abel et al., 2005; Zak et al., 2015). These inconsistent results may be explained by the different test protocols applied, study duration, dosages of L‐arginine, form of L‐arginine supplement, and also the level of physical fitness. For example, oral supplementation of L‐arginine was used in combination with various other metabolites/salts in several studies that may cause synergistic or antagonistic effects (McConell, 2007). Furthermore, the training status of the subjects seems to be an important factor related to the positive effect of LAS. LAS could have lower positive effects in well‐trained participants comparing with untrained people (Besco et al., 2012). Moreover, different L‐arginine dosages used in chronic and acute supplementation protocols could have different physiological mechanisms of action. A recent meta‐analysis reported that the effective dose of LAS should be adjusted to 0.15 g/kg body weight taken 60–90 min before exercise in the acute protocol or 10–12 g LAS for 8 weeks in chronic protocol for improving both aerobic and anaerobic performances (Viribay et al., 2020).

L‐arginine can improve exercise performance by enhancing protein synthesis and reducing muscle fiber damage (Lomonosova et al., 2014). It is also the precursor of nitric oxide that is used to increase endurance and improvement in blood flow (Alvares et al., 2011; Moncada & Higgs, 1993).One of the possible mechanisms to describe the increase in VO2 max is the nitric oxide derived from L‐arginine that results in vessel vasodilatation and flow, which, in turn, may positively influence coronary perfusion. An increase in NO production may enhance oxygen and nutrients delivery to the active muscles. Therefore, oxygen consumption increases dramatically in the active muscles with a parallel increase in muscle blood flow. (Burgomaster et al., 2006; Nagaya et al., 2001; Stamler & Meissner, 2001).

The oral LAS also facilitates the phase II pulmonary VO2 response. The proposed mechanism to explain this effect is an increase in L‐arginine availability, with subsequent increases in certain tricarboxylic acid cycle intermediates which finally lead to enhance the oxidative metabolism (Koppo et al., 2009). However, further studies are needed to understand the exact mechanisms of how L‐arginine affects VO2 max in healthy human subjects.

Although this is the first meta‐analysis that evaluates the effect of LAS on VO2 max in healthy human subjects, it has some limitations. There were some differences in the supplementation protocols, doses, timing, and also form of L‐arginine in the included trials which limited the extraction of strong conclusions.

## 5 CONCLUSIONS

This meta‐analysis indicated that LAS had a positive effect on increasing VO2 max. Future homogeneous and well‐designed randomized clinical trials are needed to a deep understand of the effects of L‐arginine on VO2 max in healthy human subjects.

## ETHICS APPROVAL AND CONSENT TO PARTICIPATE

This study has been approved by Local ethics review boards at Shahid Beheshti University of Medical Sciences.

## CONSENT FOR PUBLICATION

Institutional consent forms were used in this study.

## CONFLICT OF INTEREST

The authors declare that they have no conflict of interest.

## AUTHORS' CONTRIBUTIONS

Maryam Gholamalizadeh, Saeid Doaei, and Shahla Rezaei designed the study, and were involved in the data collection, analysis, and drafting of the manuscript. Reza Tabrizi, Peyman Nowrouzi‐Sohrabi, and Samira Rastgoo were involved in the design of the study, analysis of the data, and critically reviewed the manuscript. All authors read and approved the final manuscript.

## Data Availability

Not applicable.

## References

[phy214739-bib-0001] Abel, T. , Knechtle, B. , Perret, C. , Eser, P. , Von Arx, P. , & Knecht, H. (2005). Influence of chronic supplementation of arginine aspartate in endurance athletes on performance and substrate metabolism. International Journal of Sports Medicine, 26(05), 344–349. 10.1055/s-2004-821111 15895316

[phy214739-bib-0002] Adams, M. R. , Forsyth, C. J. , Jessup, W. , Robinson, J. , & Celermajer, D. S. (1995). Oral L‐arginine inhibits platelet aggregation but does not enhance endothelium‐dependent dilation in healthy young men. Journal of the American College of Cardiology, 26(4), 1054–1061. 10.1016/0735-1097(95)00257-9 7560599

[phy214739-bib-0003] Alvares, T. S. , Meirelles, C. M. , Bhambhani, Y. N. , Paschoalin, V. M. , & Gomes, P. S. (2011). L‐Arginine as a potential ergogenic aidin healthy subjects. Sports Medicine, 41(3), 233–248. 10.2165/11538590-000000000-00000 21395365

[phy214739-bib-0004] Besco, R. , Sureda, A. , Tur, J. A. , & Pons, A. (2012). The effect of nitric‐oxide‐related supplements on human performance. Sports Medicine, 42(2), 99–117. 10.2165/11596860-000000000-00000 22260513

[phy214739-bib-0005] Burgomaster, K. A. , Heigenhauser, G. J. , & Gibala, M. J. (2006). Effect of short‐term sprint interval training on human skeletal muscle carbohydrate metabolism during exercise and time‐trial performance. Journal of Applied Physiology, 100(6), 2041–2047. 10.1152/japplphysiol.01220.2005 16469933

[phy214739-bib-0006] Burtscher, M. , Brunner, F. , Faulhaber, M. , Hotter, B. , & Likar, R. (2005). The prolonged intake of L‐arginine‐L‐aspartate reduces blood lactate accumulation and oxygen consumption during submaximal exercise. Journal of Sports Science & Medicine, 4(3), 314. 24453536 PMC3887335

[phy214739-bib-0007] Camic, C. L. , Housh, T. J. , Mielke, M. , Zuniga, J. M. , Hendrix, C. R. , Johnson, G. O. , Schmidt, R. J. , & Housh, D. J. (2010). The effects of 4 weeks of an arginine‐based supplement on the gas exchange threshold and peak oxygen uptake. Applied Physiology, Nutrition, and Metabolism, 35(3), 286–293. 10.1139/H10-019 20555372

[phy214739-bib-0008] Campbell, B. I. , La Bounty, P. M. , & Roberts, M. (2004). The ergogenic potential of arginine. Journal of the International Society of Sports Nutrition, 1(2), 1–4. 10.1186/1550-2783-1-2-35 18500948 PMC2129157

[phy214739-bib-0009] Campbell, B. , Roberts, M. , Kerksick, C. , Wilborn, C. , Marcello, B. , Taylor, L. , Nassar, E. , Leutholtz, B. , Bowden, R. , Rasmussen, C. , Greenwood, M. , & Kreider, R. (2006). Pharmacokinetics, safety, and effects on exercise performance of L‐arginine α‐ketoglutarate in trained adult men. Nutrition, 22(9), 872–881. 10.1016/j.nut.2006.06.003 16928472

[phy214739-bib-0010] Chen, S. , Kim, W. , Henning, S. M. , Carpenter, C. L. , & Li, Z. (2010). Arginine and antioxidant supplement on performance in elderly male cyclists: A randomized controlled trial. Journal of the International Society of Sports Nutrition, 7(1), 13 10.1186/1550-2783-7-13 20331847 PMC2860344

[phy214739-bib-0011] Denis, C. , Dormois, D. , Linossier, M. , Eychenne, J. , Hauseux, P. , & Lacour, J. (1991). Effect of arginine aspartate on the exercise‐induced hyperammoniemia in humans: a two periods cross‐over trial. Archives Internationales de Physiologie, de Biochimie et de Biophysique, 99(1), 123–127. 10.3109/13813459109145914 1713484

[phy214739-bib-0012] Doaei, S. , Hajiesmaeil, M. , Aminifard, A. , Mosavi‐Jarrahi, S. , Akbari, M. , & Gholamalizadeh, M. (2018). Effects of gene polymorphisms of metabolic enzymes on the association between red and processed meat consumption and the development of colon cancer; a literature review. Journal of Nutritional Science, 7 10.1017/jns.2018.17 PMC6176493 30305892

[phy214739-bib-0013] Epstein, F. H. , Moncada, S. , & Higgs, A. (1993). The L‐arginine‐nitric oxide pathway. New England Journal of Medicine, 329(27), 2002–2012. 10.1056/NEJM199312303292706 7504210

[phy214739-bib-0014] Gutin, B. , Yin, Z. , Humphries, M. C. , & Barbeau, P. (2005). Relations of moderate and vigorous physical activity to fitness and fatness in adolescents. The American Journal of Clinical Nutrition, 81(4), 746–750. 10.1093/ajcn/81.4.746 15817847

[phy214739-bib-0015] Haghshenas, R. , Jamshidi, Z. , Doaei, S. , & Gholamalizadeh, M. (2019). The effect of a high‐intensity interval training on plasma vitamin D level in obese male adolescents. Indian Journal of Endocrinology and Metabolism, 23(1), 72–75. 10.4103/ijem.IJEM_267_18 31016157 PMC6446690

[phy214739-bib-0016] Higgins, J. (2011). Cochrane handbook for systematic reviews of interventions. Version 5.1. 0 [updated March 2011]. The Cochrane Collaboration. Retrieved from www.cochrane‐handbook.org

[phy214739-bib-0017] Hosseini, A. , & Valipour Dehnou, V. , Azizi, M. , & Khanjari Alam, M. (2015). Effect of high‐intensity interval training (HIT) for 4 weeks with and without L‐arginine supplementation on the performance of women's futsal players. Quarterly of Horizon of Medical Sciences, 21(2), 113–119. 10.18869/acadpub.hms.21.2.113

[phy214739-bib-0018] Kalman, D. , Harvey, P. D. , Perez Ojalvo, S. , & Komorowski, J. (2016). Randomized prospective double‐blind studies to evaluate the cognitive effects of inositol‐stabilized arginine silicate in healthy physically active adults. Nutrients, 8(11), 736 10.3390/nu8110736 PMC5133120 27869715

[phy214739-bib-0019] Koppo, K. , Taes, Y. E. , Pottier, A. , Boone, J. , Bouckaert, J. , & Derave, W. (2009). Dietary arginine supplementation speeds pulmonary VO2 kinetics during cycle exercise. Medicine & Science in Sports & Exercise, 41(8), 1626–1632. 10.1249/MSS.0b013e31819d81b6 19568197

[phy214739-bib-0020] Larsen, F. , Weitzberg, E. , Lundberg, J. , & Ekblom, B. (2007). Effects of dietary nitrate on oxygen cost during exercise. Acta Physiologica, 191(1), 59–66. 10.1111/j.1748-1716.2007.01713.x 17635415

[phy214739-bib-0021] Liberati, A. , Altman, D. G. , Tetzlaff, J. , Mulrow, C. , Gøtzsche, P. C. , Ioannidis, J. P. , Clarke, M. , Devereaux, P. J. , Kleijnen, J. , & Moher, D. (2009). The PRISMA statement for reporting systematic reviews and meta‐analyses of studies that evaluate health care interventions: Explanation and elaboration. Journal of Clinical Epidemiology, 62(10), e1–e34. 10.1016/j.jclinepi.2009.06.006 19631507

[phy214739-bib-0022] Lomonosova, Y. N. , Shenkman, B. S. , Kalamkarov, G. R. , Kostrominova, T. Y. , & Nemirovskaya, T. L. (2014). L‐arginine supplementation protects exercise performance and structural integrity of muscle fibers after a single bout of eccentric exercise in rats. PLoS One, 9(4), e94448 10.1371/journal.pone.0094448 24736629 PMC3988069

[phy214739-bib-0023] Mashiko, T. , Umeda, T. , Nakaji, S. , & Sugawara, K. (2004). Position related analysis of the appearance of and relationship between post‐match physical and mental fatigue in university rugby football players. British Journal of Sports Medicine, 38(5), 617–621. 10.1136/bjsm.2003.007690 15388551 PMC1724951

[phy214739-bib-0024] McConell, G. K. (2007). Effects of L‐arginine supplementation on exercise metabolism. Current Opinion in Clinical Nutrition and Metabolic Care, 10(1), 46–51. 10.1097/MCO.0b013e32801162fa 17143054

[phy214739-bib-0025] Melik, Z. , Zaletel, P. , Virtic, T. , & Cankar, K. (2017). L‐arginine as dietary supplement for improving microvascular function. Clinical Hemorheology and Microcirculation, 65(3), 205–217. 27814280 10.3233/CH-16159

[phy214739-bib-0026] Moazami, M. , Taghizadeh, V. , Ketabdar, A. , Dehbashi, M. , & Jalilpour, R. (2015). Effects of oral L‐arginine supplementation for a week, on changes in respiratory gases and blood lactate in female handballists. Iranian Journal of Nutrition Sciences & Food Technology, 9(4), 45–52.

[phy214739-bib-0027] Muazzezzaneh, A. , Keshavarz, S. A. , Sabour Yaraghi, A. A. , Djalali, M. , & Rahimi, A. (2010). Effect of L‐arginine supplementation on blood lactate level and VO2 max at anaerobic threshold performance. KAUMS Journal (FEYZ), 14(3), 200–208.

[phy214739-bib-0028] Nagaya, N. , Uematsu, M. , Oya, H. , Sato, N. , Sakamaki, F. , Kyotani, S. , Ueno, K. , Nakanishi, N. , Yamagishi, M. , & Miyatake, K. (2001). Short‐term oral administration of L‐arginine improves hemodynamics and exercise capacity in patients with precapillary pulmonary hypertension. American Journal of Respiratory and Critical Care Medicine, 163(4), 887–891. 10.1164/ajrccm.163.4.2007116 11282761

[phy214739-bib-0029] Pahlavani, N. , Entezari, M. , Nasiri, M. , Miri, A. , Rezaie, M. , Bagheri‐Bidakhavidi, M. , & Sadeghi, O. (2017). The effect of l‐arginine supplementation on body composition and performance in male athletes: A double‐blinded randomized clinical trial. European Journal of Clinical Nutrition, 71(4), 544–548. 10.1038/ejcn.2016.266 28120856

[phy214739-bib-0030] Preli, R. B. , Klein, K. P. , & Herrington, D. M. (2002). Vascular effects of dietary L‐arginine supplementation. Atherosclerosis, 162(1), 1–15. 10.1016/S0021-9150(01)00717-1 11947892

[phy214739-bib-0031] Ruiz, J. R. , Rizzo, N. S. , Hurtig‐Wennlöf, A. , Ortega, F. B. , W àrnberg, J. , & Sjöström, M. (2006). Relations of total physical activity and intensity to fitness and fatness in children: The European Youth Heart Study. The American Journal of Clinical Nutrition, 84(2), 299–303. 10.1093/ajcn/84.2.299 16895875

[phy214739-bib-0032] Schweizer, M. , & Richter, C. (1994). Nitric oxide potently and reversibly deenergizes mitochondria at low oxygen tension. Biochemical and Biophysical Research Communications, 204(1), 169–175. 10.1006/bbrc.1994.2441 7945356

[phy214739-bib-0033] Shidfar, F. , Bahrololumi, S. S. , Doaei, S. , Mohammadzadeh, A. , Gholamalizadeh, M. , & Mohammadimanesh, A. (2018). The effects of extra virgin olive oil on alanine aminotransferase, aspartate aminotransferase, and ultrasonographic indices of hepatic steatosis in nonalcoholic fatty liver disease patients undergoing low calorie diet. Canadian Journal of Gastroenterology and Hepatology, 2018, 1–7. 10.1155/2018/1053710 PMC5932499 29850450

[phy214739-bib-0034] Stamler, J. S. , & Meissner, G. (2001). Physiology of nitric oxide in skeletal muscle. Physiological Reviews, 81(1), 209–237. 10.1152/physrev.2001.81.1.209 11152758

[phy214739-bib-0035] Sunderland, K. L. , Greer, F. , & Morales, J. (2011). JOURNAL/jscr/04.02/00124278‐201103000‐00034/ENTITY_OV0312/v/2017‐07‐20T235437Z/r/image‐pngo2max and ventilatory threshold of trained cyclists are not affected by 28‐day L‐arginine supplementation. The Journal of Strength and Conditioning Research, 25(3), 833–837. 10.1519/jsc.0b013e3181c6a14d 20581700

[phy214739-bib-0036] Viribay, A. , Burgos, J. , Fernández‐Landa, J. , Seco‐Calvo, J. , & Mielgo‐Ayuso, J. (2020). Effects of arginine supplementation on athletic performance based on energy metabolism: A systematic review and meta‐analysis. Nutrients, 12(5), 1300. 10.3390/nu12051300 PMC7282262 32370176

[phy214739-bib-0037] Yaman, H. , Tiryaki‐Sönmez, G. , & Gürel, K. (2010). Effects of oral L‐arginine supplementation on vasodilation and VO2 max in male soccer players. Biomedical Human Kinetics, 2(1), 25–29. 10.2478/v10101-010-0006-x

[phy214739-bib-0038] Zak, R. B. , Camic, C. L. , Hill, E. C. , Monaghan, M. M. , Kovacs, A. J. , & Wright, G. A. (2015). Acute effects of an arginine‐based supplement on neuromuscular, ventilatory, and metabolic fatigue thresholds during cycle ergometry. Applied Physiology, Nutrition, and Metabolism, 40(4), 379–385. 10.1139/apnm-2014-0379 25781198

